# Rare Cytogenetic Anomalies in Two Pediatric Patients with Acute Leukemia

**DOI:** 10.4274/tjh.galenos.2020.2019.0425

**Published:** 2020-05-06

**Authors:** Süreyya Bozkurt, Şule Ünal, Turan Bayhan, Fatma Gümrük, Mualla Çetin

**Affiliations:** 1 Istinye University Faculty of Medicine, Department of Medical Biology, İstanbul, Turkey; 2 Hacettepe University Faculty of Medicine, Department of Pediatric Hematology, Ankara, Turkey; 3 Dr. Abdurrahman Yurtaslan Oncology Hospital, Clinic of Pediatric Oncology and Hematology, Ankara, Turkey

**Keywords:** Acute myeloid leukemia, Rare cytogenetic anomalies, Karyotype

## To the Editor,

Structural chromosomal abnormalities are frequently seen in both pediatric acute lymphoblastic leukemia (ALL) and acute myeloid leukemia (AML) cases [1,2,3]. Although some chromosomal abnormalities are common, other abnormalities are rarely seen [4,5]. In this study two relatively rare cytogenetic abnormalities are reported.

All procedures were performed in accordance with the Helsinki Declaration and approved by the local ethics committee (Approval No: GO 16/267-45).

## Case One

CALLA+ pre-B-cell ALL was diagnosed in an 8-year-old-boy. The complete blood count (CBC) at diagnosis revealed hemoglobin of 5.5 g/dL, white blood cell (WBC) count of 2.8x10^9^/L, and platelet count of 301x10^9^/L. He had t(1;4)(q42;q22) in all twenty metaphases as a sole abnormality (). The ALLIC-BFM-2009 treatment protocol was started. Bone marrow examination on day 15 revealed remission. The patient was diagnosed in 2006. The last follow-up visit was in December 2019 and he is still alive.

## Case Two

A girl of two and half months was diagnosed with the AML FAB-M5 phenotype. She had no comorbid disease and the diepoxybutane (DEB) test for Fanconi’s anemia was negative. CBC results at diagnosis revealed hemoglobin of 10 g/dL, WBC count of 9.2x10^9^/L,and platelet count of 365x10^9^/L. The AML-BFM-2004 protocol was initiated. The karyotype of the patient was 46,XX,t(1;11)(p32;q23)[19]/46,XX [1]. Bone marrow aspiration of the patient showed that she had entered the remission.

Herein, we report two rare translocations. t(1;4)(q42;q22) was found in Case 1 with ALL and this anomaly has been reported in one case to date according to the database in which we searched [6]. The previous case was also a pediatric ALL patient, as in our case [7]. While we found t(1;4)(q42;q22) as a sole abnormality in all metaphases, the anomaly was found in a complex karyotype in the previously reported case. The hybrid gene formed as a consequence of this t(1;4)(q42;q22) and its function are not known. Our case is the second reported case with this anomaly and thus contributes to the literature.

In our second case, t(1;11)(p32;q23) was found, which has been seen in a total of seven pediatric AML cases to date [6]. The ages of patients in whom this abnormality was previously detected were between 0 and 12 years, two of them being infants; our patient was 2.5 months old. When the FAB classification of the patients was examined for the previously reported cases, M0, M1, M4, and M5 were found. Hayashi et al. [8] reported this anomaly for the first time in a 7-year-old patient with AML M1 and they did not find this anomaly at diagnosis; instead, it was detected during the remission of the patient. In our case, t(1;11)(p32;q23) was present at the time of diagnosis of acute leukemia. The result of t(1;11)(p32;q23) is the *MLL-EPS15* fusion gene. The role of this fusion gene in the pathogenesis of AML is not known, but it has been suggested that the coiled-coil domains of EPS15 mediate oligomerization and activate MLL [9,10,11].

The prognostic values of rare cytogenetic anomalies are unknown. The accumulation of knowledge about rare cytogenetic anomalies detected in childhood leukemia is expected to contribute to a better understanding of the pathogenesis of these diseases.

## Figures and Tables

**Figure 1:**
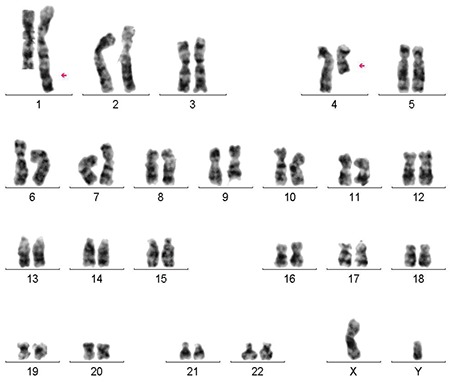
Case 1 revealed t(1;4)(q42;q22) in all twenty metaphases as a sole abnormality.
